# Predictive and Prognostic Values of Glycoprotein 96, Androgen Receptors, and Extranodal Extension in Sentinel Lymph Node-Positive Breast Cancer: An Immunohistochemical Retrospective Study

**DOI:** 10.3390/jcm13247665

**Published:** 2024-12-16

**Authors:** Tihana Klarica Gembić, Damir Grebić, Tamara Gulić, Mijo Golemac, Manuela Avirović

**Affiliations:** 1 Clinical Hospital Center Rijeka, Department of Nuclear Medicine, Krešimirova 42, 51000 Rijeka, Croatia; 2 Clinical Hospital Center Rijeka, Department of General and Oncological Surgery, Krešimirova 42, 51000 Rijeka, Croatia; damir.grebic@medri.uniri.hr; 3 Department of Surgery, Faculty of Medicine, University of Rijeka, Braće Branchetta 20, 51000 Rijeka, Croatia; 4 Department of Physiology and Immunology, Faculty of Medicine, University of Rijeka, Braće Branchetta 20, 51000 Rijeka, Croatia; tamara.gulic@medri.uniri.hr; 5 Center for Proteomics, Faculty of Medicine, University of Rijeka, Braće Branchetta 20, 51000 Rijeka, Croatia; 6 Department of General Pathology and Pathologic Anatomy, Faculty of Medicine, University of Rijeka, Braće Branchetta 20, 51000 Rijeka, Croatia; manuela.avirovic@medri.uniri.hr

**Keywords:** androgen receptors, breast neoplasms, extranodal extension, HSP90 heat shock proteins, immunohistochemistry, prognosis, sentinel lymph node

## Abstract

**Objectives**: In this paper, we investigate the association of glycoprotein 96 (GP96) and androgen receptor (AR) expression with clinicopathological factors, additional axillary lymph node burden, and their potential role in predicting 5-year overall survival (OS) and disease-free survival (DFS) in breast cancer (BC) patients with sentinel lymph node (SLN) involvement. We also explore the prognostic value of the presence of extranodal extension (ENE) in SLN. **Methods**: We retrospectively enrolled 107 female patients with cT1-T2 invasive BC and positive SLN biopsy. GP96 and AR expression were immunohistochemically evaluated on tissue microarrays constructed from two 2 mm diameter cores of formalin-fixed paraffin-embedded tumor tissues from each patient. ENE in SLN was measured in the highest (HD-ENE) and widest diameter (WD-ENE). Relative GP96 gene expression was determined using real-time quantitative PCR. **Results**: The analysis revealed ENE in SLN as the strongest predictive factor for non-SLN metastases. Patients with WD-ENE > HD-ENE had a higher risk of non-SLN metastases and worse DFS compared to those with WD-ENE ≤ HD-ENE. High GP96 expression was associated with a greater relative risk for locoregional recurrence but showed no significant impact on OS or DFS. Histological grade 3, extensive intraductal component (EIC), higher lymph node ratio (LNR), and negative AR were associated with worse DFS, while age, histological grade 3, EIC, and higher LNR were independent predictors of OS. GP96 mRNA levels were elevated in BC tissue compared to normal breast tissue. **Conclusions**: ENE in SLN is the strongest predictor of non-SLN involvement and could also have prognostic significance. While GP96 expression does not influence survival outcomes, AR expression could be used as a valuable biomarker in the follow-up of BC patients.

## 1. Introduction

Breast cancer (BC) is the most common malignancy in women and is a leading cause of female cancer mortality. Due to molecular heterogeneity, both intratumoral and intertumoral, BC continues to be a great challenge in oncology research despite remarkable achievements already made in diagnosis and treatment [1,2]. Based on the expression of estrogen (ER) and progesterone receptors (PR) and the gene amplification of the human epidermal growth factor receptor 2 (HER2), BC is classified into several molecular subtypes, each exhibiting a distinct biological molecular profile and treatment response. Molecular subtype classification is commonly used in the contemporary management of BC and plays a pivotal role in choosing therapy, thereby ensuring a precise and personalized approach to BC treatment [3,4,5,6]. More recently, the determination of androgen hormone receptor (AR) expression has provided an even wider perspective on the biological tumor profile, uncovering new therapeutic potentials [7,8,9,10,11,12,13,14]. AR belongs to the steroid hormone nuclear receptor family, sharing structural similarities with ER and PR. When inactive, AR resides in the cell cytoplasm bound to the heat shock proteins 90 (HSP90) and 70 (HSP70). Upon binding the active forms of androgen (testosterone or 5α-dihydrotestosterone), AR undergoes dimerization and translocates into the nucleus, where it initiates the regulation of target gene transcription [15]. AR influences various biological processes beyond merely the development and function of the reproductive system in both men and women. AR-mediated actions extend to bone, muscle, fat tissue, cardiovascular, immune, and neural systems, as well as hematopoiesis and coagulation [16].

In addition to its significant role in normal breast development, AR is expressed in ductal carcinoma in situ, primary BC, and metastatic BC [17]. It is overexpressed in 70–90% of BC, varying in BC molecular subtypes. AR is more frequently coexpressed in ER+ BC [18,19,20], correlating with a better prognosis and more favorable clinicopathological features. However, AR is also involved in endocrine therapy resistance in ER+ BC [19,20,21] and poor prognosis in triple-negative BC [22]. Besides prostate cancer and BC, AR signaling seems to contribute to tumor growth in several other malignancies that are not classically hormone-dependent, such as lung, renal, bladder, gastric, hepatocellular, or pancreatic cancer [23,24].

HER2 is one of the well-established prognostic and predictive biomarkers in BC. Positive HER2 status is associated with high-grade tumors, metastatic lymph node burden, higher Ki-67 proliferation index, an unfavorable response or resistance to therapy, and a poor prognosis [25]. However, with the addition of anti-HER2 targeted therapy to neoadjuvant or adjuvant systemic treatment, BC survival significantly improves [26].

Glycoprotein 96 (GP96), also known as glucose-regulated protein 94 (GRP94), plays an important role in the dimerization, activation, and downstream signaling of HER2 receptors in BC [27]. This chaperone of the HSP90 family also regulates the expression of ER-α36, involved in BC growth and proliferation [28]. High expression of GP96 correlates with more aggressive molecular BC subtypes, such as triple-negative BC, chemoresistance, and brain metastasis progression [29,30,31]. Besides its roles in cancer-promoting signaling pathways and oncogenesis, GP96 is also involved in many physiological processes and biological mechanisms essential for normal cell functioning [32]. GP96 is primarily located in the endoplasmic reticulum, where it assists in the proper folding and assembly of newly synthesized proteins, ensuring that they attain their correct conformation and functionality. It is also involved in the degradation of misfolded or improperly assembled proteins, maintaining protein homeostasis. Some of its clients include toll-like receptors, integrins, lipoprotein-receptor-related protein 6, glycoprotein A repetitions predominant, and the platelet glycoprotein Ib/IX/V, all of which play roles at various stages of cancer development, highlighting the essential role of GP96 in oncogenesis [33,34,35].

In response to cellular stressors such as hypoglycemia, acidosis, infection, or the accumulation of misfolded proteins, GP96 upregulates and translocates from the endoplasmic reticulum to the cell surface, where it engages in immune system modulation. This chaperone is also integral to the tumor microenvironment and antitumor immunity, regulating the maturation and cytotoxicity of natural killer (NK) cells, activating dendritic cells, stimulating strong T cell responses, and maintaining the function of regulatory T cells (Tregs). GP96 stabilizes and supports the function of proteins involved in immune responses and cell signaling within the tumor microenvironment, thereby increasing tumor immunogenicity [28,36,37,38,39,40,41].

Recent research highlights GP96’s potential as a promising target for cancer immunotherapy [42,43,44], and its prognostic role is still under investigation in various malignant neoplasms, such as multiple myeloma, glioblastoma, and breast, lung, pancreatic, and gastrointestinal cancers [28].

Axillary lymph node status is an important prognostic factor for BC survival, and various nomograms have been developed to predict non-sentinel lymph node (non-SLN) involvement in BC patients with SLN metastasis. Studies have identified tumor size, multifocality, lymphovascular invasion, extranodal extension (ENE) in SLN, perineural invasion, HER2 status, and Ki-67 as significant predictors of non-SLN status. While extensive research supports an association between ENE in SLN and additional non-SLN metastases, there is conflicting evidence regarding its role as a prognostic factor for BC survival [45,46].

The aim of our study is to determine the correlation of GP96 and AR expression with other clinicopathological factors and non-SLN status in early-stage BC patients with SLN involvement, as well as their impact on 5-year overall and disease-free survival (OS and DFS, respectively). We also explore the clinical significance and predictive and prognostic value of ENE in SLN.

## 2. Materials and Methods

### 2.1. Study Cohort

We retrospectively enrolled patients diagnosed with cT1-T2N0 invasive BC who underwent breast surgery and axillary lymph node dissection (ALND) following a positive SLN biopsy between January 2007 and December 2016 at our institution. All participants had newly diagnosed BC with no clinical or ultrasonographic evidence of metastases in regional lymph nodes and received phenotype-based adjuvant therapy according to the guidelines for the treatment period. Exclusion criteria were as follows: bilateral or recurrent BC, advanced-stage BC with distant metastasis at the time of diagnosis, pT3-T4 category according to TNM classification, previous breast or axillary surgery, receipt of any neoadjuvant treatment, presence of other malignancies, or non-available paraffin-embedded tumor tissue specimens. Relevant clinicopathologic and demographic data were collected from patients’ medical records and pathological reports. Positive hormone receptor (HR) status was defined as ER expression >1% and/or PR expression >1% in tumor nuclei, determined by immunohistochemical (IHC) analysis. HER2 protein expression status was determined as positive (3+) or negative (0 or 1+) by IHC staining. In cases of equivocal IHC 2+ findings, an in situ hybridization test was performed for a definitive diagnosis (32). The BC subtypes were classified into five groups according to HR, HER2, and Ki-67 proliferation index as follows: luminal A (ER+, PR+, HER2-negative, Ki-67 < 20%), luminal B HER2-negative (ER+, HER2-negative, PR-negative or <20%, and/or Ki-67 ≥ 20%), luminal B HER2-positive (ER+, any PR, HER2+, any Ki-67), HER2-enriched (ER- and PR-absent, HER2+), and triple-negative (ER- and PR-absent, HER2-negative) [47]. The presence and size of ENE in SLN were reevaluated by an experienced breast pathologist. ENE in SLN was defined as penetration of the tumor cells through the SLN capsule into the perinodal adipose tissue. ENE size was measured in two dimensions: the highest diameter (HD-ENE) and the widest diameter (WD-ENE), as described by Ma X. et al. in their research [48]. Regarding the relationship between WD-ENE and HD-ENE, we divided the patients into two subgroups, WD-ENE > HD-ENE and WD-ENE ≤ HD-ENE. Extensive intraductal component (EIC) is defined according to the College of American Pathologists (CAP) guideline recommendations [49]. Lymph node ratio (LNR) was calculated as the number of positive lymph nodes divided by the total number of removed lymph nodes [50]. The extent of axillary nodal burden in non-SLN was stratified as high if ≥2 non-SLN were found to have metastases in addition to tumor-positive SLN. Based on their age at the time of diagnosis, patients were grouped as ≤50 and >50 years old. Body mass index (BMI) was calculated as weight (kg) divided by squared height (m^2^) using self-reported or measured data and was categorized according to WHO definitions [51].

Disease recurrence included locoregional and/or distant recurrence, whichever occurred first. Locoregional recurrence (LRR) was defined as recurrence in the ipsilateral chest wall or breast or involvement of ipsilateral axillary, supraclavicular, infraclavicular, and/or internal mammary lymph nodes. A recurrence at any other site was considered a distant recurrence. Overall survival (OS) was calculated as the time from surgery to death from any cause. Disease-free survival (DFS) was defined as the time from the surgery until disease recurrence.

### 2.2. Immunohistochemical Staining and Analysis

For IHC analyses of the BC samples, we used the tissue microarray (TMA) method. Initially, representative areas within the tumor tissue were identified and marked on hematoxylin and eosin-stained (H&E) slides. These regions, representing the invasive component of the tumors, were then used to construct the TMA. Using a manual tissue microarrayer (MTA Booster OI, Alphelys, Plaisir, France), we retrieved two cylindrical cores, each measuring 2 mm in diameter, from the donor block and embedded them into a new recipient paraffin block. Subsequently, the TMA blocks were cut into serial 4 µm thick sections and transferred to adhesive glass slides. The sections were deparaffinized in a xylene substitute, rehydrated through graded alcohols, and washed with distilled water. For GP96 staining, heat-mediated antigen retrieval was performed with Tris-EDTA buffer (pH 9.0) in a water bath at 97 °C for 20 min. After blocking with 5% normal goat serum (X0907, Dako) in 1% BSA-TBS for 20 min at room temperature, the slides were incubated overnight at +4 °C in a humidified chamber with rabbit monoclonal IgG GRP94 antibody (clone D6X2Q, Cell Signalling Technology, Danvers, MA, USA) at 1:800 dilution in antibody diluent (Cell Signalling #8112). Endogenous peroxidase activity was then blocked using a peroxidase-blocking solution (S2023, Dako). Following this, the slides were incubated with goat anti-rabbit IgG secondary antibody H&L-HRP (ab6721, Abcam) at 1:500 dilution for 30 min at room temperature. Immunohistochemical visualization was performed using the EnVision Flex + system (K8000, Dako), according to the manufacturer’s instructions, with 3,3′-diaminobenzidine (DAB) as the chromogen. The sections were counterstained with hematoxylin and dehydrated through increasing concentrations of ethanol. For the negative control, diluent was used instead of the primary antibody.

Immunostained TMA was visually scored according to both the intensity of staining and the proportion of positively stained tumor cells, expressed as a percentage. The intensity score was semiquantitatively graded as follows: 0, negative; 1, weak; 2, moderate; 3, strong. An H-score was calculated by multiplying the intensity score by the proportion of positively stained tumor cells and ranged from 0 to 300. The optimal cut-off value was determined with ROC curve analysis based on patient recurrence. Tumors with an H-score >200 were considered as having high GP96 expression.

AR staining was performed using mouse monoclonal IgG anti-human AR antibody (clone AR441, Dako Cytomation, Glostrup, Denmark) at 1:100 dilution, with an automatic immunostainer (DakoAutostainer Plus; DakoCytomation, Fort Collins, CO, USA) according to the manufacturer’s instructions, as previously described [52]. Tumors with ≥10% nuclear-stained cells were defined as AR-positive.

### 2.3. Real-Time Quantitative Polymerase Chain Reaction (RT-qPCR)

Immediately after collection, fresh biopsy core tissue samples were treated with RNA Protect Tissue Reagent (Qiagen, Hilden, Germany) and stored at −80 °C until RNA isolation and analysis. TRIzol reagent (Invitrogen, Carlsbad, CA, USA) was used to extract total RNA from core biopsy tissues of luminal A, luminal B, and triple-negative BC surrogate subtypes, as well as from normal breast tissue specimens (N = 12 for each sub-group). We assessed the quality and concentration of the RNA samples by agarose gel electrophoresis (sharp 18S and 28S ribosomal RNA bands) and UV spectrophotometry (NanoDrop, Thermo Fisher Scientific, Rockford, IL, USA) (OD260/OD280 ratio). A Turbo DNA-free kit (Ambion Inc., The RNA Company, Austin, TX, USA) was used to eliminate any traces of DNA contamination. High-quality RNA samples were reverse-transcribed into cDNA using a High-Capacity cDNA Archive Kit (Applied Biosystems, Foster City, CA, USA) according to the manufacturer’s instructions. The expression levels of the GP96 gene were quantified by quantitative real-time PCR using an ABI PRISM 7300 real-time PCR system (Applied Biosystems, Foster City, CA, USA), with 18S rRNA serving as a housekeeping gene. We used commercially available TaqMan Assay reagents (Applied Biosystems) for GP96 (Hs00427665_g1) and 18S rRNA (Hs99999901_s1), following the manufacturer’s guidelines. Each procedure was independently repeated in duplicate for each sample. Relative GP96 gene levels were normalized to those of the 18S rRNA gene and expressed as 2^−ΔΔCt^ values.

### 2.4. Statistical Analysis

Statistical analysis was performed using MedCalc for Windows, version 18.2.1 (MedCalc Software, Ostend, Belgium). Patients’ clinicopathological characteristics were reported using frequencies and percentages for categorical variables or as means with standard deviations and medians with corresponding ranges for continuous variables. The differences were analyzed using the χ^2^ test, independent *t*-test, or Mann–Whitney test, appropriate to the variable type. The Kruskal–Wallis test or ANOVA were used to compare differences between groups depending on the data distribution or variable type. For the correlation analysis, we used either Spearman’s or Pearson’s correlation. Logistic regression analysis was used to evaluate relationships between clinicopathological features and non-SLN status in a univariable model. Variables showing a significant association with non-SLN status were selected for multivariable analysis. Kaplan–Meier estimates were used to analyze 5-year OS and DFS between groups, and the differences were compared using the log-rank test. Cox’s univariable and multivariable proportional hazard regression were used to analyze the prognostic significance of clinicopathological characteristics for OS and DFS, and the results were reported as hazard ratios with 95% confidence intervals. The statistical significance level was set as *p* < 0.05.

## 3. Results

### 3.1. Clinicopathological Features

A total of 107 female patients were included in this study, and their median age was 59 years (range 28–89 years). Regarding BC molecular subtype, 34 (31.77%) patients had luminal A, 36 (33.64%) had luminal B HER2-negative, 6 (5.61%) had luminal B HER-2-positive, 21 (19.63%) had HER2-enriched, and 10 (9.35%) had triple-negative BC. The mean tumor size was 2.57 ± 1.16 cm. Forty-one tumors (38.32%) were well differentiated, fifty (46.73%) were moderately differentiated, and sixteen (14.95%) were poorly differentiated. Lymphovascular invasion, necrosis, and calcifications were detected in 91 (85%), 34 (31.77%), and 45 (42.05%) patients, respectively. The clinicopathological features of the patients, along with the results of IHC for AR and GP96 and their correlation with non-SLN status, are summarized in Table 1.

### 3.2. ENE in SLN Is the Strongest Predictor of Non-SLN Status and Also Correlates with the Extent of Nodal Burden

ENE in SLN was present in 38 (35.51%) cases. Of these, 28 (73.68%) had additional metastases in non-SLN. In 69 (64.48%) patients without ENE in SLN, 28 (40.58%) had metastases in non-SLN. In the univariate logistic analysis, histological grade 3 (*p* = 0.019), the number of positive SLN (*p* = 0.039), the size of the tumor deposit in SLN (*p* = 0.001), and the presence of ENE in SLN (*p* = 0.001) were all significantly associated with non-SLN metastases. The multivariable analysis indicated that the presence of ENE in SLN (OR 2.696, 95% CI 1.051–6.914, *p* = 0.039) and the size of tumor deposit in SLN (OR 1.103, 95% CI 1.021–1.193, *p* = 0.013) were both independent predictive factors for non-SLN metastases. The presence of ENE in SLN also positively correlated with the number of positive non-SLN (r = 0.401, *p* < 0.001). Next, we aimed to explore if there was a different impact on the risk of non-SLN metastasis depending on the orientation of ENE through a subgroup analysis. When comparing patients with WD-ENE > HD-ENE to those with WD-ENE ≤ HD-ENE, we observed that the subgroup of patients with WD-ENE > HD-ENE had higher risk for non-SLN metastases (OR 3.882, 95% CI 1.391–10.838, *p* = 0.009). In addition, this subset of patients also had worse DFS (HR 3.311, 95% CI 1.048–10.459, *p* = 0.033). However, GP96 did not show an association with non-SLN status nor a higher nodal burden in non-SLN (*p* = 0.155 and *p* = 0.309, respectively). In contrast, AR showed a weak but statistically significant negative association with non-SLN status (r = −0.195, *p* = 0.044) but not with the extent of nodal burden (*p* = 0.143). Of 56 (52.33%) patients having metastases in non-SLN, 16 (28.57%) also had ENE in non-SLN, and the majority of these had ENE in SLN as well. However, five (31.25%) of the patients with ENE in non-SLN did not have ENE present in SLN. The results of the univariable and multivariable logistic regression analyses between clinicopathological variables and non-SLN status are shown in Table 2.

### 3.3. GP96 Expression Is Associated with HR Status and Recurrence in BC

**Figure 1:**
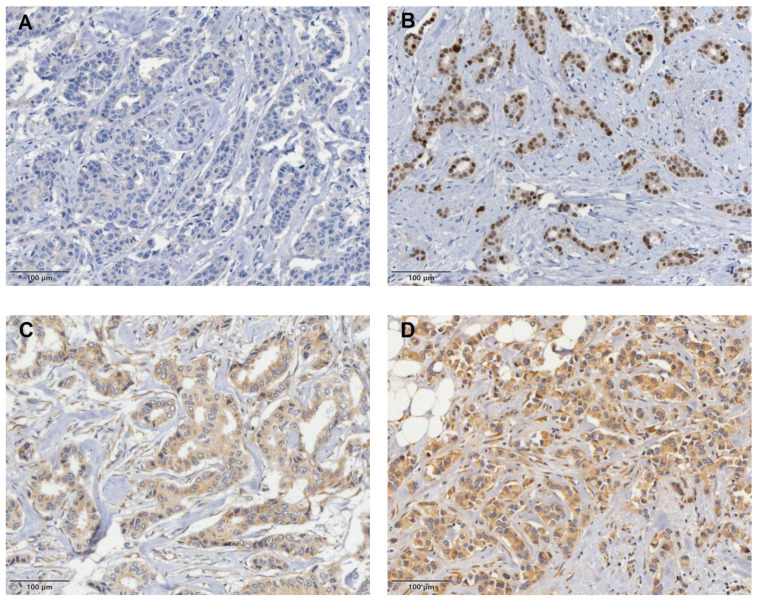

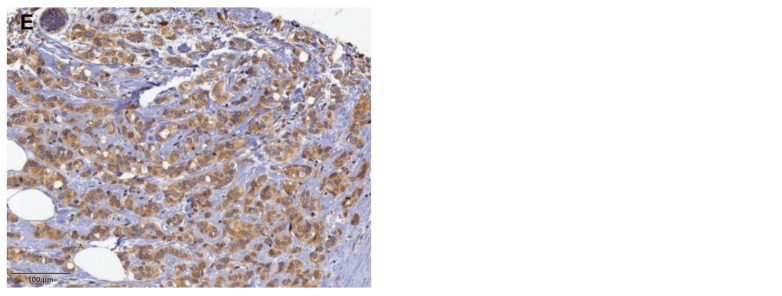
Representative images of immunohistochemical staining for androgen receptors (ARs) and glycoprotein 96 (GP96) in invasive breast cancer tissues. Negative expression of AR (**A**) and positive nuclear staining for AR (**B**). Weakly positive expression of GP96 (**C**), moderately positive expression (**D**), and strongly positive expression (**E**). Scale bar, 100 µm.

IHC analysis of GP96 expression revealed mainly cytoplasmic staining across tumor cells and some on the cell membrane, with differences in both intensity and the proportion of positive cells. The staining pattern ranged from completely negative to moderately and strongly positive. GP96+ expression was also detected in normal glandular breast tissue, tumor-infiltrating lymphocytes, and macrophages (Figure 1).

Based on the H-score, the expression of GP96 protein was classified as low in 84 (78.5%) and high in 23 (21.5%) patients, with a median GP96 expression of 165 (range 0–300). Regarding the staining intensity, GP96 expression was negative in 5 (4.67%), weak in 48 (44.86%), moderate in 31 (28.97%), and strong in 23 (21.5%) tumors. Tumors with low GP96 expression were more likely to be ER- and PR-positive, HER2-negative, and Ki-67 < 20%. There was also a statistically significant difference between luminal A and luminal B HER2-negative vs. HER2-enriched and triple-negative BC regarding GP96 protein expression (*p* < 0.001). We found a significant negative association between GP96 expression and ER (r = −0.468, *p* < 0.001) and PR status (r = −0.407, *p* < 0.001). However, no association was found between AR and GP96 expression (r = −0.167, *p* = 0.085). Further on, we wanted to investigate whether GP96 expression impacts the relative risk for locoregional or distant recurrence of the disease. The relative risk for locoregional recurrence was greater for patients with high GP96 expression compared to those with low GP96 expression (RR 4.565, CI 1.333–15.638, *p* = 0.016), but there was no significant difference for distant recurrence (RR 1.044, CI 0.380–2.868, *p* = 0.934).

### 3.4. Negative AR Expression Correlates with Higher Rates of Locoregional and Distant Recurrence in BC

AR was detected in the nucleus of tumor cells and was considered as a sign of androgen activation (Figure 1). Positive AR expression was detected in 76 (71.03%) patients, while negative AR expression was found in 31 (28.97%) patients. The median percentage of AR expression was 77 (range 0–100%). Of the 76 (71.03%) HR-positive BC cases, 62 (81.58%) were also AR-positive. Meanwhile, of the 31 (28.97%) HR-negative BC cases, 17 (54.84%) were AR-negative as well. There was no statistical difference between AR-positive and AR-negative subgroups regarding HER2 status or Ki-67 (*p* = 0.288 and *p* = 0.603, respectively). The relative risk for locoregional recurrence and distant recurrence was greater for patients in the AR-negative subgroup than for those in the AR-positive subgroup (RR 4.903, CI 1.308–18.382, *p* = 0.018, and RR 2.451, CI 1.075–5.589, *p* = 0.033, respectively). Next, we performed the subgroup analysis based on the coexpression of GP96 and AR. The groups were as follows GP96lowAR+, GP96lowAR-, GP96highAR- and Gp96highAR+. In 63 (58.88%) patients, we observed low GP96 and positive AR expression. Additionally, 21 (19.62%) patients had low GP96 and negative AR expression, 10 (9.35%) had high GP96 and negative AR expression, and 13 (12.15%) had high Gp96 and positive AR expression. The majority of HR-positive BC cases (75%) showed low GP96 and positive AR expression, while eight (25.80%) patients within the HR-negative group showed high GP96 and negative AR expression. Analysis of the subgroups based on the coexpression of GP96 and AR revealed an association with HER2 status (*p* < 0.001), ER and PR status (both *p* < 0.001), number of metastatic sites (*p* = 0.030), and BC surrogate molecular subtypes (*p* < 0.001) while showing no significant association with other clinicopathological factors. The relationship between clinicopathologic variables and GP96 and AR expression is shown in Table 3.

### 3.5. Survival Analysis

The median follow-up time was 61 months (range 15–71 months) for OS and 60 months (range 10–71 months) for DFS. Within the follow-up time, 28 (26.17%) patients died, and 22 (20.56%) experienced recurrences, of which 4 (18.18%) were locoregional only, 13 (59.09%) distant only, and 5 (22.73%) combined. Patients having one, two, three, and four or more than four different metastatic sites involved were 12 (54.55%), 2 (9.09%), 4 (18.18%), and 4 (18.18%), respectively. The most common metastatic sites were bones and lungs. Both AR and GP96 expression did not show a significant association with the number of metastatic sites (r = −0.184, *p* = 0.058 and r = 0.188, *p* = 0.052, respectively). Conversely, the presence of ENE in SLN (*p* = 0.013), negative ER status (*p* = 0.010), higher histological grade (*p* = 0.008), Ki-67 ≥20% (*p* = 0.017), higher number of positive non-SLN (*p* = 0.020), higher pN stage (*p* = 0.045), and larger metastasis size in both SLN and non-SLN (*p* = 0.011 and *p* = 0.009, respectively) were significantly associated with a higher number of metastatic sites. The presence of EIC showed a tendency toward a higher number of metastatic sites, but this was not statistically significant (*p* = 0.050).

**Figure 2:**
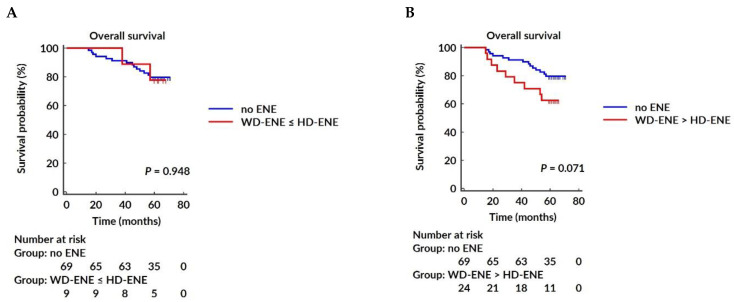

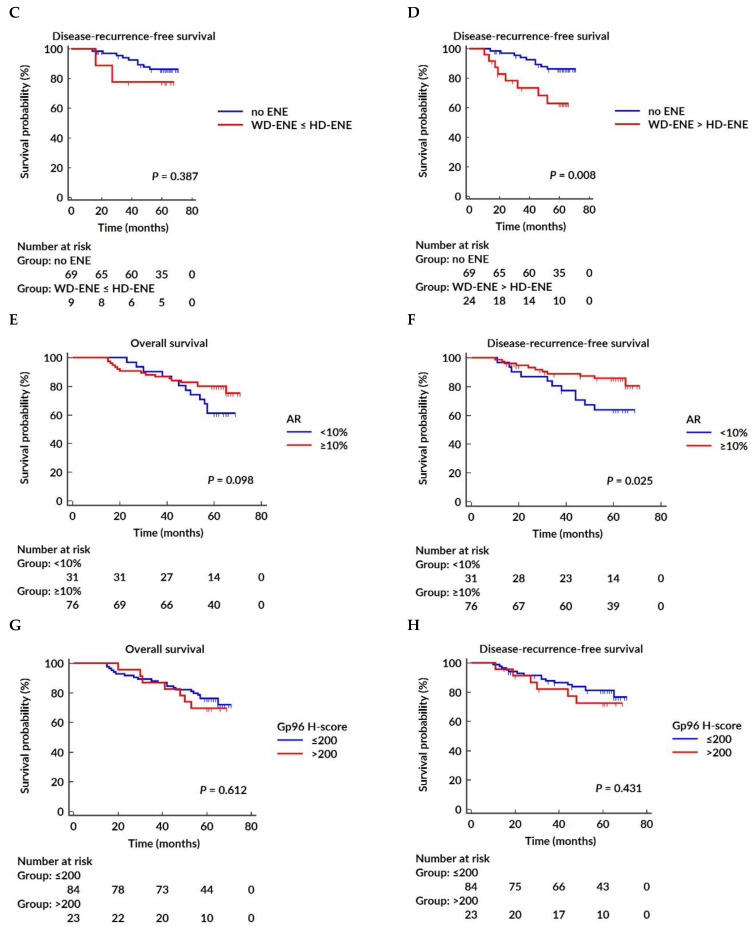
The Kaplan–Meier survival curves for overall survival (OS) and disease-free survival (DFS) according to different extranodal extension (ENE) subgroups, androgen receptor (AR) expression, and glycoprotein 96 (GP96) expression. OS survival rates comparing WD-ENE ≤ HD-ENE vs. no ENE (**A**) and WD-ENE > HD-ENE versus no ENE (**B**). DFS survival rates comparing WD-ENE ≤ HD-ENE vs. no ENE (**C**) and WD-ENE > HD-ENE vs. no ENE (**D**). OS survival rates comparing AR ≥ 10% vs. AR < 10% (**E**) and DFS survival rates comparing AR ≥ 10% vs. AR < 10% (**F**). OS survival rates comparing GP96 H-score > 200 vs. GP96 H-score ≤ 200 (**G**) and DFS survival rates comparing GP96 H-score > 200 vs. GP96 H-score ≤ 200 (**H**).

The Cox univariable proportional hazard regression analysis showed that age, histological grade, EIC, LNR, and non-SLN status were significantly associated with OS. The multivariable analysis confirmed that age, histological grade 3, presence of EIC, and LNR were independent predictors of OS, while non-SLN status was not. Neither GP96 nor AR expression showed an association with OS. For DFS, Cox’s univariable proportional hazard regression analysis identified histological grade, EIC, HR status, ENE, LNR, Ki-67, non-SLN status, and AR expression as significant prognostic factors. The multivariable analysis further demonstrated that histological grade, EIC, LNR, and AR expression were the only independent survival-associated factors for DFS. The results of Cox’s univariable and multivariable proportional hazard regression analysis for OS are presented in Table 4 and for DFS in Table 5. The Kaplan–Meier survival curves for OS and DFS, stratified by AR and GP96 expression, as well as the presence of ENE in SLN, are illustrated in Figure 2.

### 3.6. GP96 mRNA Is Elevated in BC Tissue Compared to Normal Breast Tissue

GP96 mRNA levels were significantly elevated in the luminal A, luminal B, and triple-negative BC surrogate subtypes compared to normal breast tissue (all *p* < 0.001). Notably, the triple-negative BC surrogate subtype had the highest levels of GP96 mRNA, showing a statistically significant difference when compared to both hormone-dependent BC subtypes (luminal A and luminal B, both *p* < 0.001).

## 4. Discussion

Axillary lymph node status is recognized as the leading prognostic factor for BC survival with poorer outcomes observed when lymph nodes are involved [53]. Factors such as age, tumor multifocality, location, histological grade, tumor size, and LVI and HR status are all well-established contributing factors to axillary lymph node metastatic burden [54]. Studies indicate that 30–40% of BC patients have axillary lymph node involvement [55]. Therefore, accurate axillary evaluation is an essential determinant of the pathologic stage of BC, which directly influences treatment decisions. Historically, ALND has been the gold standard for locoregional control of the invasive BC and precise axillary staging. However, since the 1990s, the SLN biopsy has become the established standard of care in managing early-stage clinically node-negative BC and has largely replaced ALND [56,57]. The National Surgical Adjuvant Breast and Bowel Project (NSABP) B-32 trial was a large-scale study involving more than 5000 women, assessing SLN biopsy versus ALND in female patients with BC. It investigated whether there are differences in survival rates, locoregional control of the disease, and morbidity between these two treatment modalities. This randomized controlled study showed that there was no statistical difference between the two cohorts in terms of survival and locoregional control, suggesting that when SLN is negative, SLN biopsy alone without further ALND is a safe, precise, and effective method providing optimal regional lymph node management, without adverse effects on survival. Furthermore, Julian et al. presented the results of a 10-year follow-up of the NSABP B-32 trial and confirmed that no significant differences in OS and DFS persisted between SLN-negative patients who had ALND or SLN biopsy alone [58]. Researchers have sought to determine whether ALND can be safely omitted in selected patients with limited axillary metastatic lymph node burden. This has led to several practice-changing clinical trials supporting the safe avoidance of ALND in selected groups of patients with early-stage BC and limited SLN involvement [59,60,61,62,63,64]. However, there is still a great deal of debate regarding the optimal management of BC patients with limited SLN involvement, as 34.3% to 77.3% are found to have non-SLN metastasis after ALND [65]. Numerous studies have evaluated clinicopathological features and different biological markers to predict non-SLN status, particularly in the context of surgical de-escalation of axillary management and avoiding the morbidity associated with the completion of ALND.

In the present study, we evaluated ENE, AR, and GP96 expression in early-stage BC patients with SLN involvement and their associations with clinicopathological features, non-SLN status, recurrence, and survival outcomes.

Previous research has established an association between the presence of ENE in SLN and a significantly increased risk of non-SLN metastases in BC patients. Ma et al. conducted retrospective research that included 402 patients with invasive BC and axillary lymph node involvement. They found that patients with ENE had higher nodal burden and higher N stage of the disease. They also explored the role of ENE in patients with different nodal stages and found that patients with N3-stage disease and ENE had worse disease-recurrence-free survival compared to those without ENE. However, they found no significant correlation between ENE and survival in N1- and N2-stage patients [48]. Choi et al. reported that patients with ENE had a significantly higher number of total positive axillary lymph nodes and non-SLN, as well as a higher frequency of the N2 stage of the disease [66]. Another study also observed that patients with ENE are more likely to have ≥4 positive axillary lymph nodes, further confirming that ENE is an independent predictor of extensive axillary nodal burden [67]. Other factors, such as younger age, higher histological grade, presence of LVI, larger tumor size, macrometastases in SLN, the number of positive and negative SLN, and HER2 overexpression, have also been suggested to influence the risk of non-SLN metastases [65,68]. In our study, 38 (35.51%) patients had ENE present in SLN, which is somewhat similar to that observed by Ma et al. (39.3%) and Yang et al. (37.6%) [48,69]. We found that histological grade, the number of positive SLN, the size of SLN metastasis, and the presence of ENE in SLN correlated with the presence of non-SLN metastases. Further multivariate analysis verified that the presence of ENE in SLN, as well as the size of the tumor deposit in SLN, were independent risk factors for non-SLN tumor burden, consistent with previous reports [70]. Nottegar et al., in their meta-analysis, also reported that ENE in SLN is one of the most important predictors of non-SLN status [71]. Meretoja et al. conducted a large-scale international multicenter study that included 675 BC patients with SLN macrometastases. They aimed to develop a predictive model for the accurate estimation of patients who are at risk of having substantial axillary tumor burden and could potentially benefit from the completion of ALND, axillary radiotherapy, or both combined [72]. Their findings also corroborate with the meta-analysis performed by van la Parra et al. [73]. Research reports have demonstrated conflicting results regarding the relationship between ENE and BC survival. Aziz et al. found that ENE >3 mm, measured perpendicularly from the lymph node capsule, was associated with worse breast-cancer-specific survival and DFS [74]. Ma et al. published a study investigating predictive and prognostic values of ENE and identifying ENE cut-off values for both perpendicular and circumferential ENE diameters (PD-ENE and CD-ENE, respectively). They found that ENE was correlated with worse disease-recurrence-free survival but not with OS. However, subgroup analysis based on determined cut-off values for PD-ENE (2 mm cut-off) and CD-ENE (3 mm cut-off) revealed no prognostic value [48]. According to Nottegar et al., the presence of ENE in SLN is strongly correlated with poor prognosis and disease recurrence [71]. Bucci et al. also observed poorer survival in patients with ENE compared to those without ENE [75]. In part, these disparities may be attributed to variations in ENE evaluation methods. The lack of standardization in evaluating and reporting ENE, along with different proposed cut-off values, varying from 1 mm to 3 mm, contributes to inconsistencies across studies, as presented by Tang et al. in their international survey [76]. Similarly to the study of Ma et al. [48], we also performed subgroup analyses regarding ENE orientation, WD-ENE, and HD-ENE, respectively. However, instead of using specific cut-off values for these parameters, we divided the patients into two subgroups, WD-ENE > HD-ENE and WD-ENE ≤ HD-ENE, respectively. The analysis showed that WD-ENE > HD-ENE was associated with a higher risk for non-SLN metastases, as well as worse DFS, but not OS. GP96 expression was not associated with non-SLN status or higher nodal burden. In contrast, AR expression was negatively associated with non-SLN involvement but showed no correlation with the extent of nodal burden.

GP96 is a member of the HSP90 family and is most abundantly located in the endoplasmic reticulum, where it maintains protein homeostasis. Hodorova et al. evaluated GP96 expression using IHC and found that GP96 is significantly more expressed in BC than in normal breast tissue [77]. A recent study also observed high GP96 expression in hepatocellular carcinoma compared to normal liver tissue [78]. The tumor microenvironment has garnered increasing attention in research due to its important roles in cancer prognosis and immunomodulation, resulting from the interaction between tumor cells and different tumor-infiltrating inflammatory cells. Previous research has demonstrated that GP96 activates both innate and adaptive immune responses, and it is essential for toll-like receptor activation and priming potent T cell response [38]. Inconsistent findings exist regarding GP96 expression and survival prognosis in different cancers.

In our cohort, 84 (78.50%) patients had a low expression of GP96 protein. This subset of patients was also more likely to have ER- and PR-positive, HER2-negative tumors with low Ki-67 (<20%). We found that GP96 expression had a negative correlation with ER and PR. Radolović et al. also reported the difference in GP96 expression regarding HR status [79]. However, no association was found with the expression of AR, and our study was the first one to explore this association. Remarkably, patients with high GP96 expression had a 4.5-fold higher risk for locoregional recurrence compared to those with low GP96 expression. In contrast, we did not observe a significant difference in distant recurrence between patient subgroups regarding GP96 expression. In concordance with our results, similar findings about the risk for locoregional recurrence have also been published [79].

We performed a survival analysis with a follow-up period of 5 years or more, and we showed that GP96 expression had no impact on OS and DFS in early-stage BC cancer. According to the literature, high GP96 expression in bladder cancer [80], gastric cancer, and esophageal cancer is associated with worse OS. However, Ji et al. found that low GP96 expression predicted poor prognosis in patients with hepatocellular carcinoma [78]. Corresponding findings were shown in a study conducted by Lee et al. in their extensive research including 709 patients with colorectal cancer [81].

AR has long been studied in prostate cancer, as it was found to be a key driver for prostate cancer development and progression. However, the complex role of AR in BC is much less clear. AR is found to exert different functions depending on the BC surrogate molecular subtype. Previous studies showed that in ER-positive BC, AR interacts with the ERα via specific binding domains, and this interaction can inhibit both of the proteins. AR can directly upregulate tumor-suppressor genes or upregulate the expression of tumor-suppressing proteins. Through both of these mechanisms, it can repress tumor growth. In this context, BC patients with both ER- and AR-positive expression have better pathohistological features and better DFS [82]. Our findings are in line with these observations. However, results from different studies investigating the association between AR in triple-negative BC surrogate molecular subtype and survival remain contradictory [80,83]. Besides the cross-talk between HR and AR, there is also evidence of AR’s involvement in HER2 signaling pathways and its role in promoting tumor cell proliferation in HER2-positive BC [80].

The tumor, node, metastasis (TNM) staging system remains the gold standard for prognostication in oncology. However, with the growing body of literature on the biological diversity of BC, the TNM staging system has evolved over the years. Several biological biomarkers, including ER, PR, HER2, histological grade, and multigene assays, have been integrated into the conventional anatomic staging system. These biomarkers significantly impact disease outcome and survival [6].

In our cohort, we identified several factors associated with worse DFS, including histological grade 3, presence of EIC, higher LNR, and negative AR expression. These factors could serve as potential prognosticators of disease recurrence in early-stage BC with SLN involvement.

Our study has some limitations we would like to address. It was a single-center, retrospective study that included a relatively small number of patients, consisting exclusively of patients with cT1-T2N0-invasive BC who underwent breast surgery and ALND following a positive SLN biopsy. The limited number of patients may have influenced the subgroup analyses. Further larger studies are needed to evaluate the clinical significance and the prognostic value of ENE in SLN, GP96, and AR in BC. Additionally, their cut-off values also warrant further investigation.

## 5. Conclusions

Our findings underscore the importance of ENE in SLN in the risk evaluation of non-SLN involvement. The presence of ENE in SLN, along with the size of SLN metastasis, is associated with a high risk of non-SLN metastasis and could have prognostic value. We also identified a subgroup of patients at higher risk of disease recurrence, defined by histological grade 3, presence of EIC, higher LNR and negative AR expression. Age, histological grade 3, EIC, and higher LNR were independent predictors of OS. While GP96 expression does not impact OS or DFS, high GP96 expression is associated with higher rates of locoregional recurrence, suggesting its potential as an additional biomarker for locoregional invasiveness in BC treatment planning and follow-up.

## Figures and Tables

**Table 1:** Clinicopathological characteristics of patients and their correlation with non-SLN status.

Characteristics (All)		No. (%)	Non-SLN Status		*p* Value
			Positive	Negative	
Entire cohort		107 (100%)	56 (52.34%)	51 (47.66%)	0.630
Age, years	median (range)	59 (28–89)	57 (34–89)	60 (28–85)	
	≤50 years	29 (27.1%)	15 (14.02%)	14 (13.08%)	0.939
	>50 years	78 (72.90%)	41 (38.32%)	37 (34.58%)	
Breast cancer laterality	left	51 (47.66%)	24 (22.43%)	27 (25.23%)	0.299
	right	56 (52.34%)	32 (29.91%)	24 (22.43%)	
Histologic type	ductal/NST	85 (79.44%)	43 (40.19%)	42 (39.25%)	0.754
	lobular	14 (13.08%)	8 (7.48%)	6 (5.61%)	
	others	8 (7.48%)	5 (4.67%)	3 (2.80%)	
Histologic grade	G1	41 (38.32%)	19 (17.76%)	22 (20.56%)	0.042
	G2	50 (46.73%)	24 (22.43%)	26 (24.30%)	
	G3	16 (14.95%)	13 (12.15%)	3 (2.80%)	
Tumor size, cm	mean ± std.	2.57 ± 1.16	2.75 ± 1.31	2.37 ± 0.94	0.087
pT stage	pT1	44 (41.12%)	21 (19.63%)	23 (21.49%)	0.427
	pT2	63 (58.88%)	35 (32.71%)	28 (26.17%)	
pN stage	1	68 (63.55%)	19 (17.76%)	49 (45.79%)	<0.001
	2	39 (36.45%)	37 (34.58%)	2 (1.87%)	
Necrosis	absent	73 (68.22%)	36 (33.65%)	37 (34.58%)	0.361
	present	34 (31.78%)	20 (18.69%)	14 (13.08%)	
Calcifications	absent	62 (57.94%)	33 (30.84%)	29 (27.10%)	0.830
	present	45 (42.06%)	23 (21.50%)	22 (20.56%)	
EIC	not evident	89 (83.18%)	47 (43.93%)	42 (39.25%)	0.829
	present	18 (16.82%)	9 (8.41%)	9 (8.41%)	
ER status	negative	31 (28.97%)	19 (17.76%)	12 (11.21%)	0.239
	positive	76 (71.03%)	37 (34.58%)	39 (36.45%)	
PR status	negative	39 (36.45%)	22 (20.56%)	17 (15.88%)	0.525
	positive	68 (63.55%)	34 (31.78%)	34 (31.78%)	
HER2 status	negative	80 (74.77%)	40 (37.38%)	40 (37.38%)	0.407
	positive	27 (25.23%)	16 (14.95%)	11 (10.29%)	
Ki-67 index	<20%	56 (52.34%)	28 (26.17%)	28 (26.17%)	0.614
	≥20%	51 (47.66%)	28 (26.17%)	23 (21.49%)	
Lymphovascular invasion	negative	16 (14.95%)	6 (5.61%)	10 (9.34%)	0.200
	positive	91 (85.05%)	50 (46.73%)	41 (38.32%)	
Perineural invasion	negative	30 (28.04%)	15 (14.02%)	15 (14.02%)	0.836
	positive	49 (45.79%)	25 (23.36%)	24 (22.43%)	
	CBD *	28 (26.17%)	16 (14.95%)	12 (11.22%)	
ENE in SLN	negative	69 (64.49%)	28 (26.17%)	41 (38.32%)	0.001
	positive	38 (35.51%)	28 (26.17%)	10 (9.34%)	
Size of SLN metastasis, mm	mean ± std.	9.19 ± 6.75	11.40 ± 6.68	6.80 ± 6.04	<0.001
AR	<10%	31 (28.97%)	17 (15.89%)	14 (13.08%)	0.742
	≥10%	76 (71.03%)	39 (36.45%)	37 (34.58%)	
GP96	low	84 (78.50%)	47 (43.93%)	37 (34.58%)	0.154
	high	23 (21.50%)	9 (8.41%)	14 (13.08%)	
BMI, kg/m^2^	≤25	48 (44.86%)	23 (21.49%)	25 (23.36%)	0.459
	>25	58 (54.21%)	32 (29.91%)	26 (24.30%)	
	N/A	1 (0.93%)			
Type of surgery	mastectomy	34 (31.78%)	24 (22.43%)	10 (9.34%)	0.010
	BCS	73 (68.22%)	32 (29.91%)	41 (38.32%)	
Molecular subtype	luminal A	34 (31.77%)	18 (16.82%)	16 (14.95%)	0.750
	luminal B/HER2-	36 (33.64%)	16 (14.95%)	20 (18.69%)	
	luminal B/HER2+	6 (5.61%)	3 (2.80%)	3 (2.80%)	
	HER2-enriched	21 (19.63%)	13 (12.15%)	8 (7.48%)	
	triple-negative	10 (9.35%)	6 (5.61%)	4 (3.74%)	

EIC—extensive intraductal component; ER—estrogen receptors; PR—progesterone receptors; HER2—human epidermal growth factor 2; ENE—extranodal extension; SLN—sentinel lymph node; AR—androgen receptors; GP96—glycoprotein 96; BMI—body mass index; NST—no special type; BCS—breast conserving surgery; N/A—not available. * Cannot be determined.

**Table 2:** Univariable and multivariable logistic regression analyses between clinicopathological variables and non-SLN status.

Characteristics (All)	Univariable Analysis		Multivariable Analysis	
	OR (95% CI)	*p* Value	OR (95% CI)	*p* Value
Age (continuous)	0.999 (0.972–1.029)	0.978		
Histologic type, special vs. ductal	1.411 (0.545–3.649)	0.478		
Histologic grade, G3 vs. G1, G2	4.837 (1.291–18.128)	0.019	3.050 (0.706–13.168)	0.0135
pT stage, pT2 vs. pT1	1.369 (0.632–2.965)	0.426		
Necrosis, present vs. absent	1.468 (0.645–3.343)	0.360		
Calcifications, present vs. absent	0.919 (0.426–1.981)	0.829		
EIC, positive vs. negative	0.894 (0.324–2.462)	0.828		
HR status, positive vs. negative	0.599 (0.256–1.404)	0.238		
HER2 status, positive vs. negative	1.455 (0.601–3.521)	0.406		
Ki-67 index, ≥20% vs. <20%	1.217 (0.569–2.605)	0.612		
LVI, positive vs. negative	2.033 (0.681–6.064)	0.204		
Perineural invasion, positive vs. negative	1.042 (0.420–2.585)	0.930		
ENE in SLN, positive vs. negative	4.100 (1.722–9.760)	0.001	2.696 (1.051–6.914)	0.039
Size of SLN metastasis, mm	1.135 (1.052–1.225)	0.001	1.103 (1.021–1.193)	0.013
No. of positive SLN	1.734 (1.029–2.921)	0.039	1.496 (0.865–2.588)	0.150
AR, ≥10% vs. <10%	0.868 (0.375–2.007)	0.741		
GP96, high vs. low	0.506 (0.197–1.298)	0.156		
BMI (kg/m^2^), >25 vs. ≤25	1.338 (0.621–2.881)	0.457		

EIC—extensive intraductal component; HR status—hormone receptor status; HER2—human epidermal growth factor 2; LVI—lymphovascular invasion; ENE—extranodal extension; SLN—sentinel lymph node; AR—androgen receptors; GP96—glycoprotein 96; BMI—body mass index; OR—odds ratio; CI—confidence interval.

**Table 3:** The relationship between clinicopathological variables and GP96 and AR expression.

Characteristics (All)	AR		*p* Value	GP96		*p* Value
	<10%	≥10%		Low	High	
	No. (%)	No. (%)		No. (%)	No. (%)	
Age, years						
≤50 years	9 (8.41%)	20 (18.69%)	0.775	22 (20.56%)	7 (6.54%)	0.686
>50 years	22 (20.56%)	56 (52.34%)		62 (57.95%)	16 (14.95%)	
BC laterality						
left	12 (11.21%)	39 (36.45%)	0.239	39 (36.45%)	12 (11.21%)	0.627
right	19 (17.76%)	37 (34.58%)		45 (42.06%)	11 (10.28%)	
Histologic type						
ductal/NST	23 (21.50%)	62 (57.95%)	0.086	65 (60.75%)	20 (18.69%)	0.372
lobular	3 (2.80%)	11 (10.28%)		13 (12.15%)	1 (0.93%)	
others	5 (4.67%)	3 (2.80%)		6 (5.61%)	2 (1.87%)	
Histologic grade						
1	12 (11.21%)	29 (27.10%)	0.676	34 (31.77%)	7 (6.54%)	0.227
2	13 (12.15%)	37 (34.58%)		40 (37.38%)	10 (9.35%)	
3	6 (5.61%)	10 (9.35%)		10 (9.35%)	6 (5.61%)	
pT stage						
pT1	11 (10.28%)	33 (30.84%)	0.451	34 (31.77%)	10 (9.35%)	0.796
pT2	20 (18.69%)	43 (40.19%)		50 (46.73%)	13 (12.15%)	
pN stage						
1	22 (20.56%)	46 (42.99%)	0.311	52 (48.60%)	16 (14.95%)	0.501
2	9 (8.41%)	30 (28.04%)		32 (29.91%)	7 (6.54%)	
Necrosis						
absent	19 (17.76%)	54 (50.47%)	0.328	59 (55.14%)	14 (13.08%)	0.395
present	12 (11.21%)	22 (20.56%)		25 (23.37%)	9 (8.41%)	
Calcifications						
absent	22 (20.56%)	40 (37.38%)	0.083	45 (42.05%)	17 (15.89%)	0.081
present	9 (8.41%)	36 (33.65%)		39 (36.45%)	6 (5.61%)	
EIC						
not evident	27 (25.24%)	62 (57.94%)	0.491	72 (67.29%)	17 (15.89%)	0.182
present	4 (3.74%)	14 (13.08%)		12 (11.21%)	6 (5.61%)	
ER status						
negative	17 (15.89%)	14 (13.08%)	<0.001	15 (14.02%)	16 (14.95%)	<0.001
positive	14 (13.08%)	62 (57.95%)		69 (64.49%)	7 (6.54%)	
PR status						
negative	19 (17.76%)	20 (18.69%)	<0.001	22 (20.56%)	17 (15.89%)	<0.001
positive	12 (11.21%)	56 (52.34%)		62 (57.94%)	6 (5.61%)	
HER2 status						
negative	21 (19.62%)	59 (55.14%)	0.288	68 (63.55%)	12 (11.21%)	0.005
positive	10 (9.35%)	17 (15.89%)		16 (14.96%)	11 (10.28%)	
Ki-67 index						
<20%	15 (14.02%)	41 (38.32%)	0.603	49 (45.80%)	7 (6.54%)	0.018
≥20%	16 (14.95%)	35 (32.71%)		35 (32.71%)	16 (14.95%)	
LVI						
negative	4 (3.74%)	12 (11.22%)	0.705	13 (12.15%)	3 (2.80%)	0.773
positive	27 (25.23%)	64 (59.81%)		71 (66.36%)	20 (18.69%)	
Perineural invasion						
negative	11 (10.28%)	19 (17.76%)	0.083	22 (20.56%)	8 (7.48%)	0.483
positive	9 (8.41%)	40 (37.38%)		41 (38.32%)	8 (7.48%)	
CBD *	11 (10.28%)	17 (15.89%)		21 (19.62%)	7 (6.54%)	
ENE in SLN						
negative	21 (19.63%)	48 (44.86%)	0.655	51 (47.67%)	18 (16.82%)	0.121
positive	10 (9.34%)	28 (26.17%)		33 (30.84%)	5 (4.67%)	
Non-SLN status						
negative	14 (13.08%)	37 (34.58%)	0.742	37 (34.58%)	14 (13.08%)	0.154
positive	17 (15.89%)	39 (36.45%)		47 (43.93%)	9 (8.41%)	
BMI, kg/m^2^						
≤25	14 (13.08%)	34 (31.78%)	0.858	35 (32.71%)	13 (12.15%)	0.223
>25	16 (14.96%)	42 (39.25%)		48 (44.86%)	10 (9.35%)	
N/A	1 (0.93%)	-		1 (0.93%)	-	
Molecular subtype						
Luminal A	6 (5.61%)	28 (26.18%)	<0.001	32 (29.91%)	2 (1.87%)	<0.001
Luminal B/HER2−	7 (6.54%)	29 (27.10%)		32 (29.91%)	4 (3.74%)	
Luminal B/HER2+	1 (0.93%)	5 (4.67%)		5 (4.67%)	1 (0.93%)	
HER2-enriched	9 (8.41%)	12 (11.21%)		11 (10.28%)	10 (9.34%)	
Triple-negative	8 (7.48%)	2 (1.87%)		4 (3.74%)	6 (5.61%)	

EIC—extensive intraductal component; ER—estrogen receptors; PR—progesterone receptors; HER2—human epidermal growth factor 2; ENE—extranodal extension; SLN—sentinel lymph node; AR—androgen receptors; GP96—glycoprotein 96; BMI—body mass index; NST—no special type; non-SLN—non-sentinel lymph node status; N/A—not available. * Cannot be determined.

**Table 4:** Cox’s univariable and multivariable proportional hazard regression analyses for overall survival.

Characteristics (All)	Overall Survival			
	Univariable Analysis		Multivariable Analysis	
	HR (95% CI)	*p* Value	HR (95% CI)	*p* Value
Age (continuous)	1.035 (1.004–1.066)	0.028	1.052 (1.020–1.085)	0.001
Histologic type, special vs. ductal	1.927 (0.871–4.263)	0.105		
Histologic grade, G3 vs. G1, G2	3.287 (1.483–7.286)	0.003	3.235 (1.257–8.330)	0.015
pT stage, pT2 vs. pT1	1.852 (0.815–4.205)	0.141		
Necrosis, present vs. absent	1.698 (0.803–3.590)	0.166		
Calcifications, present vs. absent	0.433 (0.184–1.019)	0.055		
EIC, positive vs. negative	2.383 (1.046–5.429)	0.039	2.806 (1.165–6.758)	0.021
HR status, positive vs. negative	0.522 (0.246–1.104)	0.089		
HER2 status, positive vs. negative	1.146 (0.504–2.608)	0.745		
Ki-67 index, ≥20% vs. <20%	1.849 (0.865–3.951)	0.113		
LVI, positive vs. negative	0.804 (0.305–2.120)	0.659		
Perineural invasion, positive vs. negative	0.528 (0.233–1.196)	0.126		
ENE, positive vs. negative	2.087 (0.987–4.414)	0.054		
Non-SLN status, positive vs. negative	3.339 (1.418–7.860)	0.005	1.689 (0.517–5.520)	0.386
AR, ≥10% vs. <10%	0.537 (0.254–1.136)	0.104		
GP96, high vs. low	1.247 (0.530–2.934)	0.614		
LNR (continuous)	1.032 (1.011–1.053)	0.002	1.031 (1.001–1.063)	0.044
BMI (kg/m^2^), >25 vs. ≤25	0.762 (0.358–1.621)	0.480		

EIC—extensive intraductal component; HR status—hormone receptor status; HER2—human epidermal growth factor 2; LVI—lymphovascular invasion; ENE—extranodal extension; non-SLN status—non-sentinel lymph node status; AR—androgen receptors; GP96—glycoprotein 96; BMI—body mass index; HR—hazard ratio; CI—confidence interval.

**Table 5:** Cox’s univariable and multivariable proportional hazard regression analyses for disease-recurrence-free survival.

Characteristics (All)	Disease-Recurrence-Free Survival			
	Univariable Analysis		Multivariable Analysis	
	HR (95% CI)	*p* Value	HR (95% CI)	*p* Value
Age (continuous)	1.002 (0.969–1.035)	0.928		
Histologic type, special vs. ductal	1.890 (0.769–4.645)	0.165		
Histologic grade, G3 vs. G1, G2	2.899 (1.130–7.435)	0.027	3.280 (1.218–8.837)	0.019
pT stage, pT2 vs. pT1	1.576 (0.642–3.867)	0.321		
Necrosis, present vs. absent	1.067 (0.435–2.620)	0.887		
Calcifications, present vs. absent	0.745 (0.312–1.776)	0.506		
EIC, positive vs. negative	2.743 (1.112–6.765)	0.028	2.920 (1.138–7.493)	0.026
HR status, positive vs. negative	0.384 (0.166–0.888)	0.025	0.776 (0.285–2.114)	0.620
HER2 status, positive vs. negative	1.673 (0.700–4.001)	0.247		
Ki-67 index, ≥20% vs. <20%	2.720 (1.108–6.681)	0.029	1.786 (0.659–4.842)	0.254
LVI, positive vs. negative	1.183 (0.349–4.009)	0.787		
Perineural invasion, positive vs. negative	0.806 (0.324–2.005)	0.642		
ENE, positive vs. negative	2.478 (1.058–5.806)	0.037	2.585 (0.876–7.627)	0.085
Non-SLN status, positive vs. negative	2.479 (1.009–6.091)	0.048	0.748 (0.213–2.621)	0.650
AR, ≥10% vs. <10%	0.397 (0.172–0.916)	0.030	0.304 (0.129–0.714)	0.006
GP96, high vs. low	1.454 (0.569–3.718)	0.435		
LNR (continuous)	1.033 (1.010–1.056)	0.005	1.043 (1.016–1.070)	0.001
BMI (kg/m^2^), >25 vs. ≤25	0.830 (0.360–1.914)	0.662		

EIC—extensive intraductal component; HR status—hormone receptor status; HER2—human epidermal growth factor 2; LVI—lymphovascular invasion; ENE—extranodal extension; non-SLN status—non-sentinel lymph node status; AR—androgen receptors; GP96—glycoprotein 96; BMI—body mass index; HR—hazard ratio; CI—confidence interval.

## Data Availability

The data that support the findings of this study are available on request from the corresponding author. Due to privacy and ethical restrictions, the data contain personal patient information, including names, and therefore are not publicly available.
